# Changes in attitudes toward and patterns in traditional Korean medicine among the general population in South Korea: a comparison between 2008 and 2011

**DOI:** 10.1186/1472-6882-14-436

**Published:** 2014-11-07

**Authors:** Jong-Min Woo, Eun-Ji Park, Minhee Lee, Miyoung Ahn, Soohyun Kwon, Ki Hoon Koo

**Affiliations:** Policy Division, Korea Institute of Oriental Medicine, 1672 Yuseongdae-ro, Yuseong-gu, Daejeon 305-811 South Korea; Herbal Medicine Research Division, Korea Institute of Oriental Medicine, 1672 Yuseongdae-ro, Yuseong-gu, Daejeon 305-811 South Korea

**Keywords:** Traditional Korean medicine, Attitude, South Korea, Prevalence, 2011

## Abstract

**Background:**

Traditional Korean medicine (TKM) is acknowledged to be prevalent among the Korean public, but few follow-up studies are available to confirm this commonly held belief. Whereas most survey studies have focused on the demographic factors influencing the usage of TKM, only a few studies have conducted a pattern or trend analysis over time. The purpose of this paper is to observe and document recent trends in the usage of TKM in South Korea and to compare overall patterns of TKM use over a period of several years.

**Methods:**

A cross-sectional survey was conducted in 2011 to assess TKM usage patterns and public perceptions regarding TKM. An online questionnaire was administered to consenting respondents that focused upon individual preferences between TKM and current Western medicine, respondents’ reasons for using TKM, the frequency of respondents’ visits to TKM clinics, the reasons respondents visited TKM clinics, and respondents’ perceived satisfaction.

**Results:**

The results revealed that 66.6% of the respondents showed a positive attitude toward TKM. In addition, 69.3% of the respondents had visited TKM clinics one to four times during the previous year. Patients used TKM with the intentions of receiving acupuncture (95.3%), moxibustion (40.1%), and cupping (36.0%) treatments or to take herbal medicines (35.7%). Most respondents who had visited TKM clinics were largely satisfied with the clinics’ effectiveness (56.1%). The factors most commonly associated with TKM usage included sex (female), age (50s), and education (college or higher), but the within-factor differences were not significant. Compared with a previous survey of other groups, TKM usage was found to have increased from 45.8% in 2008 to 69.3% in 2011. With the exception of acupuncture and physical therapy, most usage doubled or more than doubled.

**Conclusions:**

The attitudes toward and usage of TKM in South Korea have improved between 2008 and 2011. This result will be used to explain outcomes of certain social phenomena and to argue for national support in the promotion of TKM.

**Electronic supplementary material:**

The online version of this article (doi:10.1186/1472-6882-14-436) contains supplementary material, which is available to authorized users.

## Background

Complementary and alternative medicine (CAM) is widely used by both healthy people and patients with various medical problems in many countries. Nearly half of the population in many industrialized countries, including the U.S., Australia, France, Canada, and Japan, commonly use CAM therapies, and CAM usage is also increasing in many developing countries, including China, Chile, Colombia, and Taiwan [1–3]. Thus, studies aiming to identify the factors influencing the use of CAM have been conducted in many countries. The findings from these studies correlate CAM usage with age, race, gender, education, ethnicity, income, geographic residence, disease state/health, employment, spiritual/religious philosophies, the availability of health insurance, and sexual orientation [4, 5].

CAM consists of a large and heterogeneous array of techniques and includes both therapeutic and diagnostic approaches [6]. Therefore, it is difficult to clarify or consolidate its definition, although many efforts have been made to define CAM in terms of concepts and models. Depending on an author’s perspective, the number of CAM therapies can range from four to 16 [7]. The National Center for Complementary and Alternative Medicine (NCCAM) defines CAM as a group of diverse medical and health care systems, practices, and products that are not currently considered conventional medicine [4, 8, 9]. As so defined, CAM has a broad range of implications.

TKM, as a subset of CAM, is a popular source of health care in South Korea that has been passed down from generation to generation for centuries [10] as an integral part of common cultural practices and beliefs. Whereas Western medicine is built on reproducible experiments and statistical analyses [11], TKM is based on ancestral experience. Currently, Koreans are showing considerable interest in TKM, which is reflected in a substantial financial market for TKM. Since the time when TKM was first covered by national health insurance, its usage rate has increased significantly [12]. The majority of TKM users report that they have received various services to treat diseases or to use as secondary treatments while receiving conventional care. Positive perceptions of the effectiveness of TKM are a major determining factor in its use.

The aim of this study was to improve our understanding of the general perceptions of TKM by illustrating users’ characteristics, TKM usage frequency, and users’ preferences relating to these therapies by examining data from 2011. In addition, this study sought to gain insight into developing trends in TKM consumption by comparing the data employed in this study with earlier surveys conducted by other groups.

## Methods

### Questionnaire

This study was approved by the ethical review committee of the Korea Institute of Oriental Medicine (KIOM), located in South Korea. Participants who agreed to complete the survey first provided their informed consent. The survey instrument was designed to include questions regarding TKM utilization, self-perception, types of CAM use, reasons for choosing TKM, and the frequency with which the respondent visited TKM clinics. To assess TKM usage (as opposed to CAM usage) and to ensure comparability with the 2008 survey conducted by Shin *et al*. [12] (referred to herein as the “2008 survey”), we adjusted the questionnaire to contain the following TKM modalities: acupuncture, moxibustion, cupping, physical therapy, herbal medicine, *Chuna* (including chiropracty and any type of manual therapy primarily designed to address or relieve back pain), and other modalities based on the 2008 survey [12]. Non-TKM therapies that are still considered CAM were excluded from this study, including treatments based on vitamins, honey, compound nutrient pills, meditation, and breathing exercises. To ensure the accuracy of the data that were collected, certain questions were designed to allow multiple answers (Additional file 1).

### Participants

The survey was administered via Macromill Embrain (http://www.embrain.com), one of the largest survey companies in South Korea, which was engaging 1,010,000 panelists at the time of our survey. We designed the survey with a target sample of 1,000 of these panelists by applying proportional quota sampling to those asked to participate in the study. We conducted our web-based survey between September 17 and December 8, 2011. Eligibility was limited to those who were 20 years of age or older. Among the respondents, 499 (49.9%) were male, and 501 (50.1%) were female. The participants were distributed across the country in five large metropolitan cities in South Korea: Seoul, Daejeon, Daegu, Pusan, and Gwangju.

### Comparison of the 2008 survey with our 2011 survey

To minimize heterogeneity between the 2008 survey and our survey, we had numerous meetings with Dr. HK Shin, the main author of the 2008 survey, in which we asked for advice in designing the survey and in analyzing the data. Dr. Shin has been working for our institute, KIOM, and is interested in our survey because it was the first cross-sectional survey conducted since 2008. The questionnaire that we used contained nearly the same checklists as his survey, and we followed his protocols as much as possible. In addition, the socio-demographic ratios among the participants, the age distributions of the participants, and the dates of the year in our survey were similar to those same features in the 2008 survey [12]. Because our survey instruments were mostly determined by the 2008 survey, we did not evaluate validity and reliability. However, we consulted with some experts about whether the questions were clear, understandable, and presented in a logical order prior to conducting the survey.

### Statistical analysis

All data collected in the survey were analyzed using SAS 9.3 (SAS Institute Inc.). The data were assessed using Pearson’s *chi*-square test to compare categorical variables between TKM use and the socio-demographic characteristics of the respondents. Student’s *t*-test was applied to assess the continuous variables.

## Results

### Reliability of TKM compared with western medicine

**Figure 1:**
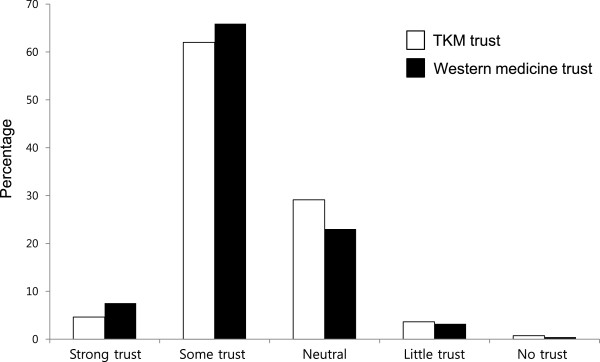
**Public opinion regarding the question** **“How much do you trust TKM and Western medicine?”** The majority of the participants trusted TKM as much as they did Western medicine for treating diseases.

A total of 1,000 participants were included in this study. The socio-demographic characteristics of the survey samples are shown in Table 1. The participants’ ages ranged from 20 to 69. Figure 1 demonstrates that the majority of the participants trusted both TKM and Western medicine. There was no significant difference with respect to gender. However, people aged 50 to 59 reported higher rates of TKM reliability (75.7%) compared with older and younger people. Notably, there was a slight positive trend in the tendency to trust TKM as the participants’ ages increased, at the following rates: 61.8% of those in their 20s; 63.3% of those in their 30s; 66.7% of those in their 40s; and 75.5% of those in their 50s. Similarly, there was a positive trend in the respondents’ favorable attitudes toward Western medicine as the participants’ age increased, at the following rates: 69.6% of those in their 20s; 72.2% of those in their 30s; 74.0% of those in their 40s; and 77.7% of those in their 50s. The reliability of TKM was perceived to be highest among those in their 50s who were college graduates and residents of Seoul. The participants who expressed trust in TKM also tended to place strong trust in Western medicine (85.6%).

**Table 1:** **Demographics of the respondents and the reliability of TKM and Western medicine**

Characteristics	Total no.	TKM reliability (%)				Western medicine reliability (%)			
		Trust ^*^	Neutral	Distrust ^†^	***P***-value	Trust	Neutral	Distrust	***P***-value
Gender					0.515				0.373
(Average)		(66.6)	(29.1)	(4.3)		(73.4)	(23.0)	(3.6)	
Male	499	66.5	28.5	5.0		75.2	21.8	3.0	
Female	501	66.7	29.7	3.6		71.7	24.2	4.2	
Age (yrs)					0.029				0.419
20-29	207	61.8	30.9	7.2		69.6	27.1	3.4	
30-39	237	63.3	33.8	3.0		72.2	24.5	3.4	
40-49	231	66.7	28.6	4.8		74.0	20.3	5.6	
50-59	206	75.7	22.3	1.9		77.7	19.9	2.4	
60-69	119	65.5	29.4	5.0		73.9	23.5	2.5	
Level of education					0.005				0.098
High school graduate	220	61.4	35.0	3.6		66.8	27.7	5.5	
College	657	70.2	26.0	3.8		75.6	21.2	3.2	
College graduate	123	56.9	35.0	8.1		73.2	24.4	2.4	
Region of residence					0.475				0.006
Seoul	541	68.8	26.6	4.6		75.8	22.0	2.2	
Daejeon	79	62.0	31.6	6.3		73.4	16.5	10.1	
Daegu	125	67.2	31.2	1.6		66.4	30.4	3.2	
Pusan	182	64.8	31.3	3.8		69.2	25.8	4.9	
Gwangju	73	58.9	35.6	5.5		78.1	17.8	4.1	
TKM reliability					<0.001				<0.001
Trust	666					85.6	13.7	0.8	
Neutral	291					52.2	40.5	7.2	
Distrust	43					27.9	48.8	23.3	
Western medicine reliability					<0.001				<0.001
Trust	734	77.7	20.7	1.6					
Neutral	230	39.6	51.3	9.1					
Distrust	36	13.9	58.3	27.8					

^*^The percentage of trust was the sum of “strong trust” and “some trust”.

^†^The percentage of distrust was the sum of “no trust” and “little trust”.

### Reasons for trusting TKM therapies

The survey asked about the participants’ reasons for trusting TKM therapies. Because several reasons were predicted, the question was structured to allow for multiple answers. The results indicated that participants’ primary reason for trusting TKM was because it has been proven useful by its utilization for hundreds of years (58.0%) or because it had been proven safe because of a low rate of side effects (52.7%). The other reasons were effectiveness (35.2%), comfort (24.6%), and strong reputation (15.7%). A small percentage of respondents felt that TKM was reliable because doctors had explained it well to them (7.9%) or because information on TKM has become more generally available to the public (4.8%) (data not shown).

### Frequency and type of TKM therapy usage

Of the respondents, 69.3% had visited TKM clinics at least once during the past 12 months. More than half (53.4%) of the general population visited TKM clinics anywhere from one to four times. Moreover, 6.6% of the general population had used TKM therapies more than 10 times in one year. Visits to TKM clinics were not correlated with any demographic factors. Only small exceptions appeared with age. The percentages of respondents who had visited TKM clinics more than 10 times were 5.8% for those in their 20s, 4.6% for those in their 30s, and 5.2% for those in their 40s, whereas those percentages were increased approximately two-fold for patients in their 60s (10.9%) (Table 2). Of the TKM clinic visitors, the majority received therapeutic treatments such as acupuncture, cupping, and moxibustion (67.7%), whereas 18.6% took traditional herbal medicines. Notably, even the respondents who did not particularly trust TKM also showed a tendency to take traditional herbal medicines and/or to receive health consultations at TKM clinics (Table 3).

**Table 2:** **Visit frequency for TKM clinics during the past 12 months**

Category	Non-user (%)	User (%)				***P***-value
		Total	No. of TKM visits			
			1-4	5-9	≥10	
Gender						0.004
(Average)	(30.7)	(69.3)	(53.4)	(9.3)	(6.6)	
Male	34.7	65.3	51.5	9.4	4.4	
Female	26.7	73.3	55.3	9.2	8.8	
Age (yrs)						0.054
20-29	38.6	61.4	49.8	5.8	5.8	
30-39	28.3	71.7	55.7	11.4	4.6	
40-49	32.5	67.5	52.4	10.0	5.2	
50-59	25.7	74.3	57.3	8.3	8.7	
60-69	26.9	73.1	50.4	11.8	10.9	
Level of education						0.211
High school graduate	33.2	66.8	51.8	5.9	9.1	
College	29.8	70.2	54.5	9.7	5.9	
College graduate	30.9	69.1	50.4	13.0	5.7	
Region of residence						0.305
Seoul	31.2	68.8	51.9	9.2	7.6	
Daejeon	31.6	65.4	53.2	8.9	6.3	
Daegu	28.8	71.2	48.8	16.0	6.4	
Pusan	30.8	69.2	57.7	7.1	4.4	
Gwangju	28.8	71.2	61.6	4.1	5.5	
TKM reliability						<0.001
Trust	25.2	74.8	54.8	11.6	8.4	
Neutral	39.9	60.1	52.6	4.8	2.7	
Distrust	53.5	46.5	37.2	4.7	4.7	
Western medicine reliability						0.038
Trust	28.1	71.9	54.8	9.9	7.2	
Neutral	37.0	63.0	51.7	7.0	4.3	
Distrust	44.4	55.6	36.1	11.1	8.3

**Table 3:** **Reasons for visiting TKM clinics**

Category	To receive therapeutic treatments (%)	To receive traditional herbal medicines (%)	Health consultations (%)	To be vaccinated (%)	To receive physical therapy (%)	***P***-value
Gender						0.655
(Average)	(67.7)	(18.6)	(13.1)	(0.3)	(0.15)	
Male	69.0	16.9	13.8	0.3	0.0	
Female	66.5	20.4	12.5	0.3	0.3	
Age (yrs)						0.158
20-29	66.9	18.1	15.0	0.0	0.0	
30-39	65.3	20.0	13.5	1.2	0.0	
40-49	62.8	22.4	14.1	0.0	0.6	
50-59	67.3	20.3	12.4	0.0	0.0	
60-69	82.8	8.0	9.2	0.0	0.0	
Level of education						0.659
High school graduate	70.1	17.7	10.9	0.7	0.7	
College	67.2	18.9	13.7	0.2	0.0	
College graduate	65.9	20.0	14.1	0.0	0.0	
Frequency of TKM clinics						<0.001
1-4	63.3	21.3	15.0	0.4	0.0	
5-9	79.6	11.8	8.6	0.0	0.0	
10 or more	86.4	7.6	4.5	0.0	1.5	
TKM reliability						0.025
Trust	72.5	17.1	10.0	0.2	0.2	
Neutral	56.6	21.7	21.1	0.6	0.0	
Distrust	45.0	35.0	20.0	0.0	0.0	

*Note*. A total of 693 individuals who underwent TKM therapies were examined.

### Usage of TKM therapies

Table 4 summarizes individual preferences regarding the types of TKM therapies. Because many CAM users report that they prefer using combined therapies over singular therapies [8], this part of the questionnaire was designed for multiple answers. The most popular therapy was acupuncture (95.3%), followed by moxibustion (40.1%), cupping (36.0%), herbal medicine (35.8%), *Chuna* (10.9%), and physical therapy (0.4%). Regardless of gender, age, educational level, or TKM reliability, most TKM users had received acupuncture treatment. The moxibustion users were predominantly in their 40s and 60s. Taking traditional Korean herbal medicine was more common among seniors (24.7% of those in their 20s compared with 43.1% of those in their 60s).

**Table 4:** **Prevalence of the use of TKM therapies**

Category	Acupuncture (%)	Moxibustion (%)	Cupping (%)	Traditional herbal medicine (%)	Chuna* (%)	Physical Therapy (%)	Others ^†^ (%)
Gender							
(Average)	(95.3)	(40.1)	(36.0)	(35.7)	(10.9)	(0.4)	(0.2)
Male	96.0	39.6	35.1	33.3	12.0	0.4	0.4
Female	94.7	40.6	36.9	38.1	9.8	0.4	0.0
Age (yrs)							
20-29	91.8	30.6	28.2	24.7	14.1	0.0	0.0
30-39	96.4	34.2	36.9	36.9	11.7	0.0	0.0
40-49	95.9	49.0	39.8	34.7	9.2	0.0	0.0
50-59	97.1	40.8	39.8	39.8	8.7	1.9	0.0
60-69	94.4	47.2	33.3	43.1	11.1	0.0	1.4
Level of education							
High school graduate	95.1	38.8	27.2	37.9	5.8	0.0	0.0
College	95.2	40.0	38.1	33.9	12.6	0.6	0.3
College graduate	96.4	42.9	41.1	42.9	10.7	0.0	0.0
TKM reliability							
Trust	96.4	41.8	39.1	38.0	10.2	0.6	0.3
Neutral	90.9	36.4	28.3	29.3	14.1	0.0	0.0
Distrust	100.0	11.1	0.0	22.2	0.0	0.0	0.0

*Note*. A total of 693 individuals who underwent TKM therapies were examined. The questions allowed for multiple answers.

*Chuna may involve chiropracty, *Chuna*, and other similar manual therapies primarily designed for back pain.

^†^Others consisted of only hot springs therapy.

### Comparison of previous TKM surveys in South Korea

**Figure 2:**
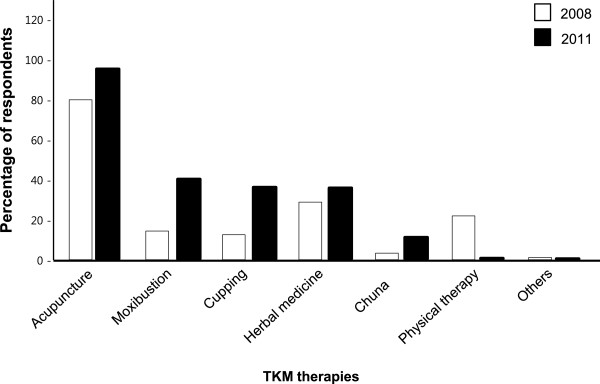
**TKM therapy use**. The graph shows the percentage of respondents who have used TKM clinics for various therapies. The 2008 survey data are obtained from Shin *et al*. [12]. *Chuna* may involve chiropracty, *Chuna*, and other similar manual therapies that largely target back pain.

Previous publications were used to compare the changes in TKM usage in South Korea over time. Three major TKM public surveys were conducted in 1999 [13], 2006 [14], and 2008 [12]. However, there was an enormous difference in the prevalence of TKM users between 1999 and 2006, when the proportion of the population using TKM jumped from 29% to 74.8%, as shown in Table 5. This disparity, however, was shown to have resulted from different definitions of TKM and the different modalities applied in each survey. These surveys also covered and included several non-traditional Korean therapies as CAM rather than TKM, including Ayurveda, music and art therapy, and dietary supplements such as vitamins and minerals. Because of the disparities in these reports, it is therefore impossible to clearly identify trends in TKM usage by comparing these particular surveys. For this reason, we considered the 2008 survey by Shin *et al*. as the first and possibly the only study to examine the usage of TKM, in particular [12]. Compared with the 2008 survey, we found that the overall percentage of TKM use had increased by 2011. In both surveys, most respondents were equally likely to receive acupuncture (80.15% in 2008 and 95.3% in 2011). The largest increases in usage involved moxibustion (21.9% in 2008 and 40.1% in 2011), cupping (12.45% in 2008 versus 36.0% in 2011), and *Chuna* (3.3% in 2008 and 10.9% in 2011), whereas the percentage engaging in TKM physical therapy significantly decreased from 21.9% in 2008 to 0.4% in 2011 (Figure 2).

**Table 5:** **TKM and**/**or CAM surveys conducted among the general public in South Korea**

Year	TKM or CAM	Age (yrs)	No. of modalities included in the questionnaire	Corresponding usage rate	References
1999	CAM	≥18	31	29%	[13]
2006	CAM	30-69	27	74.8%	[14]
2008	TKM	20-69	7	45.8%	[12]
2011	TKM	20-69	7	69.3%	

*Note. TKM* = traditional Korean medicine; *CAM* = complementary and alternative medicine. *TKM* is a subset of *CAM* that originated from Korean traditional knowledge and literature.

## Discussion

In the present study, we aimed to investigate the frequency, types, and factors associated with TKM usage among South Koreans. Although the entire population is covered by medical insurance and many people were familiar with various modalities as the major form of health care [15], others were opposed to TKM because of the lack of scientific evidence regarding its practices, adverse effects resulting from treatment, or because of the financial burden [5, 12]. Nevertheless, we found that two-thirds of our sample population used some type of TKM therapies in 2011. In 1999, the first national survey on TKM use among the general population was conducted. The results indicated that 29% of the population had used TKM within the preceding 12-month period. The out-of-pocket expenditures for CAM had grown to 40.8% of those for Western medical services [13, 16]. The next survey was conducted in 2006. The results indicated that 75% of adults reported some type of TKM therapy during the past year, for which the median annual out of-pocket expenditures were approximately US $203.6 per person [14]. Ock *et al*. suggested that the sudden increase in TKM users might be explained by the different definitions of TKM used in the two surveys. Similar to CAM [17], TKM encompasses a broad spectrum of practices and modalities. It was assumed that different age distributions were another reason for this difference. The authors had excluded individuals in their 20s (20–29 years) from the sample population. Because younger people are less inclined to use TKM as frequently than older people, these studies were likely to report higher proportions compared to other surveys. However, it is certain that the frequency of TKM use has steadily increased over the past 15 years. In this study, TKM use was highly prevalent among older adults. Compared with younger adults, older adults were more likely to visit TKM clinics to cure diseases, receive healthcare, or improve their physical and emotional well-being. As in many other countries, acupuncture was the most commonly used TKM therapy and was associated with the highest reported frequency of improvement. Acupuncture is one of the few CAM therapies that has the advantage of being accepted as modern medicine, most likely because of the volume of scientific evidence supporting it [18, 19]. Notably, TKM was preferred by women more than men in South Korea. According to the 2006 survey by Ock *et al*., females (80.3%) used TKM more commonly than males (69.3%) [14]. In the 2008 survey, more females (53.6%) used TKM than males (46.4%) did [12]. Consistent with previous results, we found that more females (75.3%) than males (65.3%) had positive attitudes toward TKM therapies. Women are also frequent users of CAM in many countries [20–22]. Nevertheless, it is difficult to obtain a comprehensive explanation as to why women prefer TKM. The usage of *Chuna* or physical therapies was found to be less prevalent among the Korean public. In general, the public could not easily distinguish *Chuna* – a type of TKM therapy largely used for balancing orthopedic structure and function – from other types of manual therapies [23]. Externally, this practice has a strong similarity to chiropracty. Furthermore, some TKM clinics are performing both of these therapies without informing the patient which therapy is being employed. When educational level is considered with respect to usage of TKM, college graduates were shown more likely to trust Western medicine (73.2%) than TKM (56.9%). However, this result does not coincide with the frequency of TKM use. In CAM surveys in developed Western countries, the educational level of CAM users was reported to be higher than that of non-users [17, 24–27]. However, other studies have reported that the educational level of CAM users was lower than that of non-users [28] or that educational level was not correlated with CAM usage at all [29, 30]. Therefore, the influence of education level on CAM and TKM usage is ambiguous and debatable. Most participants likely trusted TKM because they recognized that TKM includes therapies that have been proven useful over centuries (58.0%) and have been proven to be safe (52.7%) and effective (35.2%).

The year 2008 was considered to be a milestone year for TKM researchers because several significant aspects revealed themselves, including scientific, sociological, and political factors. First, the TKM scientific paradigm has shifted greatly and systemically. TKM research can be categorized as follows: a beginning period (before 1993), a fetal period (1993–1997), a toddler period (1998–2007), and a growth period (after 2008). Until the toddler period, most TKM researchers devoted their efforts to verifying TKM efficacies based on traditional knowledge and literature. Some of these studies were complicated, but most were small and fragmented. Since the beginning of the growth period, the notion of TKM research was expanded as a substitute for Western medicine through the discovery of alternative efficacies, mechanisms of new action, and novel substances as part of a large-scale project. Second, in terms of sociological aspects, the popularity of TKM has resulted in an increase in TKM-related professions and facilities. Korea maintains a dual licensing system of medical doctors: one for conventional Western medical doctors (MDs), and the other for traditional Korean medicine doctors (TKMDs), often called Oriental medical doctors (OMDs). During the period between the surveys (2008–2011), the number of TKMDs increased by 13.5% (17,541 in 2008 versus 19,912 in 2011), and the number of traditional Korean pharmacologists (TKPs) increased by 35.4% (1,213 in 2008 versus 1,643 in 2011). More than 20,000 TKMDs were predicted to be licensed by mid-2012, corresponding to one TKMD per 2,500 persons in South Korea. In addition, familiarity with TKM has grown because of the prevalence of TKM facilities. There was a 28.7% increase in the number of TKM hospitals (143 in 2008 versus 184 in 2011) and an 11.5% increase in the number of TKM clinics (11,135 in 2008 versus 12,421 in 2011) from 2008 to 2011. Seven hospitals specializing in TKM were also built during this period. Third, the government has promoted policies emphasizing the development of TKM by enacting laws, establishing national projects and long-term plans, and investing substantial amounts of research funds to encourage TKM practices. In 2008, the Korean government announced the “Second Basic Plan for National Science and Technology Development”, which was the first national plan to promote TKM. Also in 2008, there was strong consensus among government departments in connection with the launch of the “Long-Term Growth and Development of TKM R&D” to promote clinical studies and manufacturing. Accordingly, US $280 million were invested in TKM studies over the span of a decade. In 2009, the National Assembly amended the Medical Service Law to permit patients to undergo cooperative treatments with TKM and Western medical care in hospitals. In 2011, 128 local hospitals indicated that they offered a combination of TKM and Western medical care. This result is important because it clearly shows that attitudes toward TKM have changed to become more positive, perhaps because of the various efforts described above.

Our survey has several limitations, one of which is a geographical limitation. We collected our data from individuals living in five large metropolitan cities in South Korea, distributed evenly across the country: Seoul, Daejeon, Daegu, Pusan, and Gwangju. Given that this survey was the first cross-sectional study since the 2008 survey, some differences between the two surveys were found. First, Shin *et al*. combined two different survey methods, with the majority conducted online and a minority conducted as face-to-face interviews, whereas in the current study, we employed a single online survey. Second, the size of their sample was double the size of ours. Third, the author of the 2008 survey was focused on the occurrence of adverse events in TKM. Taken together, these differences may have limited the precision of the estimations in the study [12], and, because this study was a retrospective survey, it has the potential for recall bias. The results may also have been affected by selection bias. In addition, because we did not test the validity and reliability of our instrument, our data may contain inaccurate results. However, we tried our best to minimize possible errors/biases from the questionnaire via numerous meetings with Dr. HK Shin [12]. Nevertheless, this study is meaningful because it is the first to assess defined TKM usage by excluding non-traditional Korean modalities according to socio-demographic characteristics. Furthermore, this study is the first attempt to analyze changing trends in TKM consumption by combining its analysis and data with data from previous surveys.

## Conclusions

In this study, we used data from 2011 to identify the factors that determine TKM usage. Additionally, this study revealed that the attitude toward and usage of TKM have improved between 2008 and 2011. We suggest that the popularity of TKM can be attributed to various factors, including a paradigm shift in scientific fields, an expansion of TKM infrastructure, and unwavering support from the government since 2008. The findings are valuable when interpreting the outcomes of social phenomena and constitute a good source for demonstrating the importance and necessity of national support to promote TKM.

## Electronic supplementary material

Additional file 1: **Questionnaire on use of TKM therapy and frequency of TKM clinics.** (DOCX 19 KB)

## Abbreviations

TKM: Traditional Korean medicine
CAM: Complementary and alternative medicine
NCCAM: National Center for Complementary and Alternative Medicine
M.D: Western medical doctor
TKMD: Traditional Korean medicine doctor
OMD: Oriental medical doctor
R & D: Research and development.
