# Supplementary material for: Changes in attitudes toward and patterns in traditional Korean medicine among the general population in South Korea: a comparison between 2008 and 2011
Source: BMC Complement Altern Med. 2014 Nov 7;14:436. doi: 10.1186/1472-6882-14-436 (PMC4233081; doi:10.1186/1472-6882-14-436)
Supplement: Supplementary file 1 — Additional file 1: Questionnaire on use of TKM therapy and frequency of TKM clinics. (DOCX 19 KB) [file 12906_2014_2004_MOESM1_ESM.docx]

*Please note that this questionnaire is translated from the original Korean version.*

**Questionnaire to investigate the attitude toward Traditional Korean Medicine among the public in South Korea**

We thank you for taking the time to fill out this questionnaire. Your answers and comments will provide us with a greater understanding of the Traditional Korean Medicine (TKM) trend and help to develop a more effective scientific planning. This questionnaire is approved by the ethical review committee of the Korea Institute of Oriental Medicine, South Korea.

**Definitions:**

***Traditional Korean Medicines*** refers to traditional medical field developed and practiced in Korea.

***Acupuncture*** is the stimulation of specific points along the skin of the body involving various methods such as the application of heat, pressure, or laser or penetration of thin needles.

***Cupping*** involves warming the air inside a glass, metal, or wooden cup and inverting it over a part of the body to treat various health conditions.

Moxibustion involves the burning of mugwort, a small, spongy herb, to facilitate healing.

***Chiropractic*** is a health care profession that focuses on disorders of the musculoskeletal system and the nervous system, and the effects of these disorders on general health.

***Physical therapy*** is a health care profession primarily concerned with the remediation of impairments and disabilities and the promotion of mobility, functional ability, quality of life and movement potential through examination, evaluation, diagnosis and physical intervention.

***Traditional herbal medicines*** include herbs, herbal materials, herbal preparations and finished herbal products that contain as active ingredients parts of plants, or other plant materials, or combinations.

Please read and answer the following questions.

**Demographic and other Characteristics**

1. Gender: □ Male □ Female

2. Age (in years): □ 20-29 □ 30-39 □ 40-49 □ 50-59 □ 60-69

3. Marital status: □ Single □ Married □ Divorced

4. Educational level: □ High school graduated □ College □ College graduated

5. Residence: □ Seoul □ Daejeon □ Daegu □ Pusan □ Gwangju

6. Monthly Income: □ Less than 200 KW □ 200-500 KW □ Greater than 500 KW

7. Employment: □ Full time □ Part time □ Homemaker □ Retired □ Unemployed

8. Are you still currently working? □ Yes □ No

**TKM reliability:**

9. How much do you trust TKM?

□ A lot of □ Some □ Neutral □ Little □ No

**If little or no, skip to Question 10**

10. Why do you trust TKM therapies?

□ Proven cures for hundreds of years

□ Low rate of side effects

□ High reputation

□ Comfort

□ Effectiveness

□ Doctor’s good explanation

□ Opened TKM information to the public

□ Other (please specify) …………………

11. How much do you trust Western medicine?

□ A lot of □ Some □ Neutral □ Little □ No

**Reason for visit:**

12. How often during the last year have you visited TKM clinics?

□ Never

□ 1 to 4 times

□ 5 to 9 times

□ 10 or more

**If never, you do not need to continue. Thank you for answering this questionnaire.**

13. Which TKM therapy have you used? Please select all that apply

□ Acupuncture

□ Cupping

□ Moxibustion

□ Chiropractic

□ Physical therapy

□ Traditional herbal medicines

□ Other (please specify) …………………

14. Why did you decide to use TKM therapies? Please select all that apply

□ To get health consultation

□ To take physical therapy

□ To take traditional herbal medicine

□ To receive therapeutic treatments

□ To vaccinate

□ Other (please specify)…………………

15. Who recommended you the use of TKM therapies you had taken at first?

□ Medical doctor

□ Parents or friends

□ Media (TV/Newspaper)

□ Pharmacist

□ Other (please specify)…………………

**Others:**

16. Have you ever experienced any adverse effect from TKM therapies?

□ Yes □ No □ Don’t know

17. Can you estimate the average cost per year of TKM?

□ ( kw)

18. Which part of TKM should be focused on promoting development?

□ Evidence-based effectiveness

□ Evidence-based safeness

□ Development of new TKM products

□ Generalization of TKM therapeutic approaches

□ Theories of TKM

□ Other (please specify) …………………

**Thank you for your participation in this survey.**
